# Chromosome inheritance and meiotic stability in allopolyploid *Brassica napus*

**DOI:** 10.1093/g3journal/jkaa011

**Published:** 2020-12-22

**Authors:** Zhiyong Xiong, Robert T Gaeta, Patrick P Edger, Yao Cao, Kanglu Zhao, Siqi Zhang, J Chris Pires

**Affiliations:** 1 Key Laboratory of Herbage and Endemic Crop Biotechnology, Ministry of Education, College of Life Science, Inner Mongolia University, Hohhot, Inner Mongolia 010021, PR China; 2 Division of Biological Sciences, University of Missouri, Columbia, MO 65211, USA; 3 Bayer’s Crop Science Division, Chesterfield, MO 63017, USA; 4 Department of Horticulture, Michigan State University, East Lansing, MI 48823, USA

**Keywords:** homoeologous recombination, meiosis error, allopolyploid, polysomic inheritance, double reduction

## Abstract

Homoeologous recombination, aneuploidy, and other genetic changes are common in resynthesized allopolyploid *Brassica napus*. In contrast, the chromosomes of cultivars have long been considered to be meiotically stable. To gain a better understanding of the underlying mechanisms leading to stabilization in the allopolyploid, the behavior of chromosomes during meiosis can be compared by unambiguous chromosome identification between resynthesized and natural *B. napus*. Compared with natural *B. napus*, resynthesized lines show high rates of nonhomologous centromere association, homoeologous recombination leading to translocation, homoeologous chromosome replacement, and association and breakage of 45S rDNA loci. In both natural and resynthesized *B. napus*, we observed low rates of univalents, A–C bivalents, and early sister chromatid separations. Reciprocal homoeologous chromosome exchanges and double reductions were photographed for the first time in meiotic telophase I. Meiotic errors were non-uniformly distributed across the genome in resynthesized *B. napus*, and in particular homoeologs sharing synteny along their entire length exhibited multivalents at diakinesis and polysomic inheritance at telophase I. Natural *B. napus* appeared to resolve meiotic errors mainly by suppressing homoeologous pairing, resolving nonhomologous centromere associations and 45S rDNA associations before diakinesis, and reducing homoeologous cross-overs.

## Introduction

Although our knowledge about polyploidy has increased in recent decades, the mechanisms leading to genome stability in neopolyploids remain elusive (Ramsey and Schemske 2002; Soltis *et al.* 2004, 2010; Doyle *et al.* 2008). Meiotic instability has been observed along with novel genetic and phenotypic variation in newly formed allopolyploids [reviewed by Soltis and Soltis (1995), Doyle *et al.* (2008), and Flagel and Wendel (2010)]. The genomes of polyploids undergo a suite of changes involving reductions in chromosome and gene number, chromosome fusions, rearrangements, and the acquisition or maintenance of meiotic stability (Song *et al.* 1995; Pires *et al.* 2004; Comai 2005; Mandakova *et al.* 2010; Schubert and Lysak 2011; Matsushita *et al.* 2012). Meiotic stability must be an essential component of polyploidy evolution in sexual species and is necessary for the production of successful gametes.

*Brassica napus* (AACC; 2*n *=* *38) is a recent allopolyploid (5000–10,000 years ago) formed by the hybridization of *B. rapa* (AA; 2*n* = 20) and *B. oleracea* (CC; 2*n* = 18) (Nagaharu 1935; Chalhoub *et al.* 2014). *Brassica napus* allopolyploids can be easily resynthesized from these diploid progenitors, permitting the analysis of early genomic changes (Song *et al.* 1995; Pires *et al.* 2004; Gaeta *et al.* 2007; Książczyk *et al.* 2011; Rousseau-Gueutin *et al.* 2017). In a population of 50 resynthesized *B. napus* allopolyploids, genome-wide molecular markers and chromosome-specific fluorescent *in situ* hybridization (FISH) on mitotic cells revealed that genetic changes were common and often resulted from homoeologous rearrangement (Gaeta *et al.* 2007; Xiong *et al.* 2011). Recent studies revealed that homoeologous exchange is a major cause of gene presence/absence variation in newly resynthesized *B. napus* (Samans *et al.* 2017; Hurgobin *et al.* 2018; Schiessl *et al.* 2019). Interestingly, these previous studies showed that such chromosomal variations are non-uniformly distributed across the genome and are mainly observed in highly syntenic chromosomes (Gaeta *et al.* 2007; Xiong *et al.* 2011; Nicolas *et al.* 2012; Samans *et al.* 2017; Hurgobin *et al.* 2018).

The parental subgenomes of *B. napus* cultivars are reported to have remained largely intact (Parkin *et al.* 1995; Rana *et al.* 2004; Snowdon 2007; Cheung *et al.* 2009), and homoeologous recombination and resulting translocations are rare (Parkin *et al.* 1995, 2005; Osborn *et al.* 2003a; Piquemal *et al.* 2005; Udall *et al.* 2005; Howell *et al.* 2008). Genome resequencing of *B. napus* cultivars has detected some rearrangements between the A and C subgenomes (Chalhoub *et al.* 2014; Samans *et al.* 2017; Hurgobin *et al.* 2018); however, the frequency of observed homoeologous exchanges in these cultivars is significantly lower than what has been documented in resynthesized lines (Chalhoub *et al.* 2014).

Meiosis in polyploids has been investigated for a long time, and independent studies have reported on “meiotic irregularities” in resynthesized polyploids (Newton and Darlington 1929; Madlung *et al.* 2005; Lim *et al.* 2008; Szadkowski *et al.* 2010; Lloyd and Bomblies 2016). In *Arabidopsis* allopolyploids, chromosome fragments and bridges were observed in meiotic cells using FISH with centromere probes (Madlung *et al.* 2005). Meiotic analysis in the newly synthesized *Tragopogon* allotetraploids using GISH revealed the frequent occurrence of multivalents, suggesting homoeologous pairing (Lim *et al.* 2008; Chester *et al.* 2012). Using BAC-FISH that resulted in “GISH like” pattern on A and C chromosomes, frequent meiotic errors observed in meiosis I were inferred to be the main drivers of genome instability in resynthesized *B. napus* (Szadkowski *et al.* 2010, 2011). Although there have been extensive historic studies on meiosis in polyploids, further understanding of the mechanisms leading to stabilized allopolyploid is enabled through unambiguously chromosome identification and analysis of their meiotic behavior. Here, we report on a detailed analysis of meiosis I pairing and segregation among the 38 chromosomes of resynthesized and natural *B. napus* using a robust FISH approach.

## Materials and methods

### Plant materials

Resynthesized *B. napus* allopolyploid lines (CCAA) were developed by hybridizing doubled haploid *Brassica oleracea* line TO1000 (egg donor; C-genome) with doubled haploid *Brassica rapa* line IMB218 (pollen donor; A-genome) as described previously (Gaeta *et al.* 2007). Two replicate plants of resynthesized line EL500 were analyzed at all stages of meiosis in both of S_1_ and S_11_ generations. Line EL1200 and EL5200 at S_1_ generation was also used for detecting homoeologous rearrangement and segregation irregularities at meiotic telophase I. Natural *B. napus* doubled haploid cultivars that were selected included Stellar, Yudal, Darmor (derived from the UK *Brassica* diversity set; Darmor GT080910), and DH12075. Detailed summaries of plant materials used for this study and the number of pollen mother cells (PMCs) detected at each meiotic stage are shown in Supplementary Table S1. Since aneuploids are particularly common in resynthesized polyploid lines, several plants of each genotype were planted and karyotyped, and only the plants with additive chromosome sets and without detectable pre-existing chromosomal translocations (determined by mitotic FISH) were selected for meiotic chromosome analysis. All the plants were grown in a greenhouse environment under a 16-h-light/8-h-night photoperiod.

### Probes for FISH, tissue preparation, hybridization, and imaging

Karyotyping was performed on meiotic PMCs at prophase I, diakinesis and telophase I in both resynthesized and natural *B. napus*. Probes used for FISH, tissue preparation, hybridization, karyotyping, and imaging were described previously (Xiong and Pires 2011; Xiong *et al.* 2011). Briefly, karyotyping was performed with two probe mixtures (mixture 1 and mixture 2). Mixture 1 contained 5S rDNA and BAC clone KBrB072L17 labeled with Fluorescein-12-dUTP, 5S rDNA and BAC clone KBrH092N24 labeled with Cy3-dCTP, and 45S rDNA labeled with Cy5-dCTP. After the first round hybridization, the used slides were stripped by washing with 2× SSC containing 70% formamide at 70°C for 2 min and dehydration by dipping the slides in 95% alcohol. Then, the slides were reprobed with mixture 2, which included: Fluorescein labeled CentBr1, Cy3- labeled BAC clone BNIH123L05, and Cy5- labeled CentBr2. Together, these hybridizations allowed identification of all chromosomes and theirs corresponding homoeologs at meiotic diakinesis and telophase I in *B. napus* (Xiong and Pires 2011).

For analysis of meiosis, karyotyping using probe mixtures 1 and 2 was performed on both natural *B. napus* and three resynthesized lines. However, because extensive chromosome association occurred in diakinesis of resynthesized *B. napus* in the S_1_ generation, unambiguous identification of all chromosomes was difficult using these mixtures. As a result, for EL500 in the S_1_ generation, an additional experiment using C-genomic-specific and 15 chromosome-specific BACs (Supplementary Table S2) as probes was carried out on meiotic diakinesis chromosomes to identify homoeologous chromosome pairing (Xiong and Pires 2011). This BAC-FISH method allowed for greater resolution since the C-genomic-specific BAC to differentiate chromosomes from C- genome and chromosome-specific BAC to distinguish homoeologous chromosome pairs. Note that both methods are capable of identifying all chromosomes. Using mixtures 1 and 2 as probes, karyotype analyses were conducted on PMC at telophase I in both resynthesized and natural *B. napus*. Visualization was performed using an Olympus BX61 fluorescent microscope with the 60× plan apo oil immersion lens, and digital images were captured using the Olympus Microsuite TM 5 software package.

#### Analysis of meiotic behavior

At the stage of diakinesis, the main types of chromosome associations can be (1) univalent, where the chromosome is not associated with any other from either a homologous or homoeologous genome, (2) bivalent (or A–C bivalent), where two homologous (or homoeologous) chromosomes are paired together and not associated with any other chromosomes, (3) tri-, tetravalent and/or multivalent where more than two apparent chromosomes are associated by 45S rDNA and centromere sequences, or among homoeologous chromosomes.

### Statistical analysis

One hundred PMCs at meiotic diakinesis or telophase I were analyzed for each replicate S_1_ plant from resynthesized line EL500 and EL1200. For the S_11_ generation, 100 cells from EL500 were analyzed for one replicate, and 65 cells were analyzed for the second replicate. One hundred cells were analyzed in both replicate plants of Stellar in diakinesis. For analysis of telophase I, 50 pairs of cells were karyotyped in each replicate plant (two replicates at each generation in resynthesized lines, and one plant was analyzed for each of the four cultivars). Where appropriate, replicates were averaged for statistical analysis. Statistics were performed using data analysis functions in Excel and correlations, tests of proportions, and Mann–Whitney *U*-tests were conducted using R statistical software.

### Data availability

Reagent and data are available upon request.

Supplementary material is available at figshare DOI: https://doi.org/10.25387/g3.13056365.

## Results

### Homoeologous chromosome pairing and non-homologous centromere association during pachytene in *B. napus*

**Figure 1:**
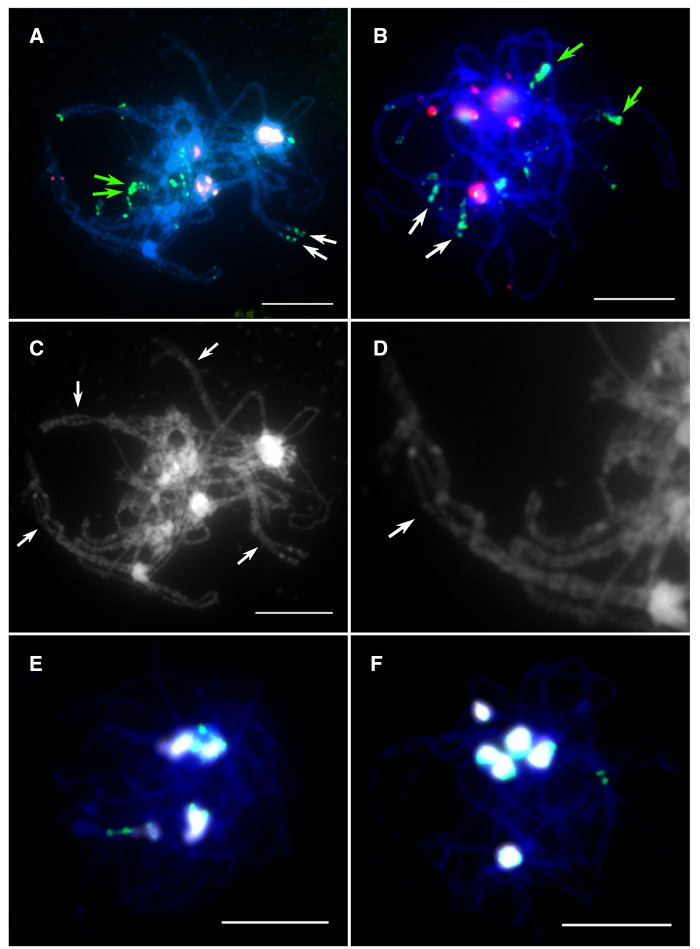
Homoeologous pairing and nonhomologous centromere association in pachytene of resynthesized and natural cultivar *Brassica napus.* (A) Two different homoeologous A–C tetravalents identified according to strength of green signals are indicated with green and white arrows, respectively. (B) Tetravalents were not detected in Stellar, and the same homoeologous sets paired in (A) are unpaired in this example [compare to green and white arrows in (A)]. (C) A gray scale image of the photo shown in (A). (D) An enlargement of a homoeologous pair shown in (A, C). Note that homologous chromosomes are not completely synapsed and pairing partner switches are observed, and that several areas show association of homoeologous chromosomes A2 and C2 (arrow) since it contains red signals from BAC clone KBrH092N24. (E) Nonhomologous centromere associations in resynthesized *B. napus* detected with a probe mixture containing centromere probes. When centromere probes (CentBr1 and CentBr2) were applied to pachynema in resynthesized *B. napus*, few, large foci were detected. (F) Nonhomologous centromere association in *B. napus* Stellar. When centromere probes (CentBr1 and CentBr2) were applied to pachynema in Stellar, few, large foci were detected (bright foci). Scale bar = 10 μm.

In resynthesized *B. napus*, homologous chromosomes predominantly formed bivalents, but parallel alignment (pairing) of homoeologous chromosomes was also observed at pachytene. Karyotype analysis of resynthesized *B. napus* line EL500 revealed that four of six cells in a single S_1_ plant and four of eight in S_11_ plants contained at least one clear tetravalent (Figure 1A). The high base chromosome number in *B. napus* made it challenging to visualize every chromosome in pachytene. Chromosomes that paired to form tetravalents were inferred to be homoeologous (Xiong *et al.* 2011). In *B. napus* cultivar Stellar, few clear tetravalents were observed (2 of 21 cells) (Figure 1B). Close-up analysis of homoeologous tetravalents in resynthesized lines revealed sections of chromosomes that failed to synapse with their homolog, and in these regions pairing partner switch between homoeologs was visible (Figure 1, C and D). These results demonstrated that early in prophase I (pachytene here), homoeologous chromosome pairing was largely suppressed in natural *B. nap*us line Stellar compared with those in resynthesized *B. napus*. When centromere probes were applied to pachynema in resynthesized *B. napus* and Stellar, few, large foci were detected (Figure 1, E and F). An average of 5.8 centromere foci were detected among 25 cells of resynthesized *B. napus*, and an average of 6.5 foci were detected among 32 cells of Stellar. Resynthesized lines and natural *B. napus* Stellar each have seven pairs of 45S rDNA loci and five pairs of 5S rDNA loci, which localize on the same set of chromosomes. Associations among 45S rDNA loci formed 2–3 large 45S rDNA signals at pachytene and were observed both in resynthesized *B. napus* (Figure 1A, white signals) and Stellar.

### Chromosome behavior during diakinesis in *B. napus*

**Figure 2:**
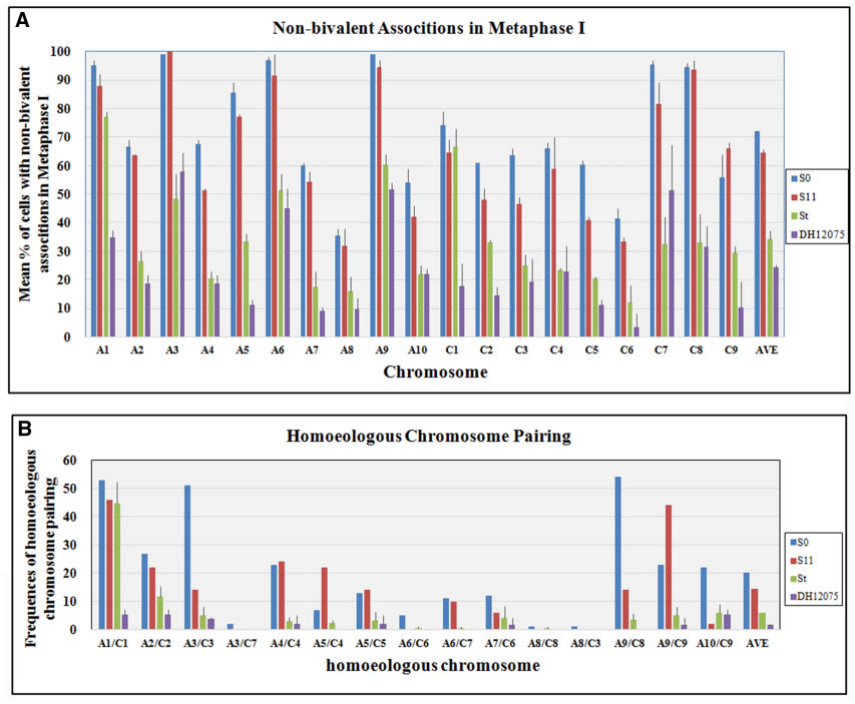
Nonhomologous associations detected during diakinesis in resynthesized and natural *Brassica napus*. (A) The average number of nonhomologous associations in resynthesized and natural *B. napus* indicated by bar height, and error bars represent standard deviations. Resynthesized lines were analyzed in the S_1_ and S_11_ generations (blue and red bars, respectively), and natural *B. napus* Stellar and DH12075 are shown with green and purple bars, respectively. The average number of nonhomologous associations across all chromosomes is summarized at the far right of the graph. (B) Homoeologous associations were analyzed from the data set presented in (A). Bar height represents the average percentage of homoeologous associations, and error bars represent standard deviations.

**Figure 3:**
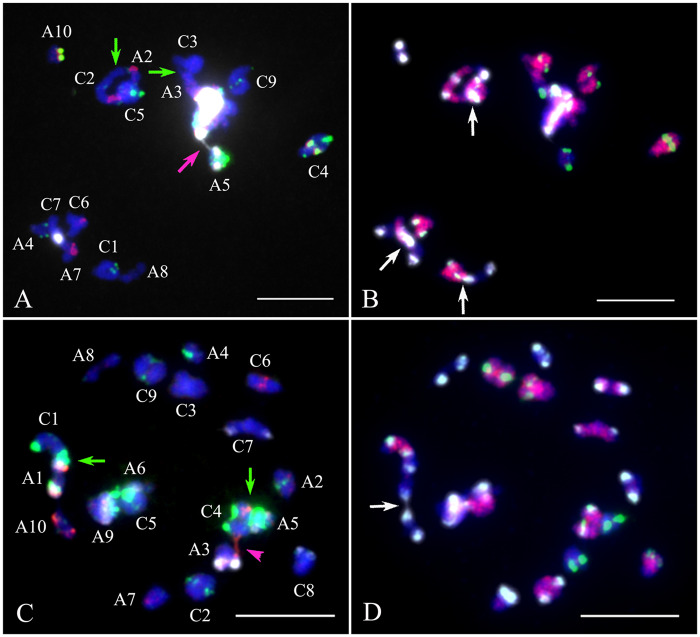
Nonhomologous chromosome association during diakinesis of *Brassica napus*. Hybridizations were first performed using probe mixture 1 (panels A and C) and probe mixture 2 (panels B and D) allowing unambiguous identification of all parental chromosomes (see *Materials and methods*). (A) Analysis of resynthesized *B. napus* revealed bivalents among homologs (*e.g.* A10, C4, C9), associations among homoeologs (green arrows; *i.e.* A2–C2, A3–C3), and association of 45S rDNA locus-containing chromosomes, which connect by a huge white signal from 45S rDNA probe (magenta arrow). (B) The same cell as in (A) reprobed with mixture 2 shows association of nonhomologous centromeres (white arrows). (C) Analysis of Stellar revealed normal association among homologs (*e.g.* A2, A4, C8, etc.), association among homoeologs (*i.e.* A1–C1 and A5–C4; green arrows), association among 45S rDNA locus-containing chromosomes (A6 and A9) and 5S containing chromosomes (A3 and C4, magenta arrowhead). (D) The same cell as in (C) reprobed with mixture 2 revealed nonhomologous centromere association by white CentBr1 signals (white arrows). Note: Each pair of homologs is denoted with a capital letter for the parental genome and the chromosome number. Scale bar = 10 μm.

In resynthesized *B. napus* and natural cultivars Stellar and DH12075, non-homologous associations (association among homoeologs, nonhomologous 45S rDNA locus-containing chromosomes, and nonhomologous centromere association) were observed during diakinesis (Figures 2A and 3). The mean number of nonhomologous associations was normally distributed among chromosomes for resynthesized *B. napus* line EL500 at both the S_1_ and S_11_ generations (Shapiro–Wilk test; *P* > 0.05) but was not normally distributed among chromosomes in *B. napus* Stellar (*P* = 0.022). The average rate of nonhomologous association among all chromosomes was not significantly different between the S_1_ (72%) and S_11_ (65%) generations (*P* = 0.276; *t*-test); however, the rate of nonhomologous pairing in both generations was significantly greater than that observed in Stellar (34%) (*P* < 0.001; Mann–Whitney *U*-test; Bonferroni correction).

Many associations observed during diakinesis occurred among homoeologous chromosomes (Figures 2B and 3). The rate of homoeologous associations was not normally distributed among chromosomes in S_1_ and S_11_ resynthesized lines, or in Stellar (Shapiro–Wilk; *P* < 0.01 for each distribution). We also rejected the null hypotheses that the counts for each chromosome came from a uniform distribution (chi-square, *P* < 0.0001 for each distribution). Counts across chromosomes were significantly correlated among the three data sets (Figure 2B; Spearman Rank correlations with Bonferroni correction, *P* < 0.01; Supplementary Table S2). The highest rates of homoeologous pairing in resynthesized *B. napus* were observed for homoeologous pairs A1–C1, A2–C2, A3–C3, A9–C8, and A9–C9. Homoeologous chromosome pairing in *B. napus* Stellar occurred among a few chromosomes, while homoeologous pairing in resynthesized EL500 *B. napus* was observed across most chromosomes (Figure 2B). In Stellar, homoeologous pairing between chromosomes A1 and C1 was observed in an average of 44.5% of cells, similar to the rates observed in resynthesized *B. napus* across generations (49–57%; Figure 2B). However, the ratio of homoeologous chromosome pairing between chromosomes A1 and C1 in *B. napus* of DH12075 was much lower than those in Stellar (Figure 2B). The average frequency of homoeologous pairing among all sets was not significantly different between the S_1_ (20%) and S_11_ (16%) generations (*P* = 0.430; Mann–Whitney *U*-test). The rates of homoeologous pairing in both generations were greater than those observed in Stellar (6%) and DH12705 (1.8%), but the difference was only significantly lower in comparison to the S_1_ generation (*P* < 0.05; Mann–Whitney *U*-test; Bonferroni correction). Our analysis of diakinesis found that overall most chromosome sets in both resynthesized and natural *B. napus* paired exclusively with their homologs, while a few demonstrated frequent homoeologous tetravalent pairing as summarized in the section above. Meanwhile, homoeologous A–C bivalents or univalents were rarely observed (Figure 2 and Supplementary Figure S1). A–C bivalent pairing between homoeologs in resynthesized lines was observed in an average of 4% of cells in the S_1_ generation (two A1–C1 and two A2–C2) and 3% of cells in the S_11_ generation (A1–C1, A2–C2, and A4–C4), and 2% cells contained homoeologous bivalents (one A1–C1 and one A2–C2) in one replicate of Stellar (Supplementary Figure S1). Cells containing univalent A and/or C chromosomes were infrequent and were observed at similar rates in resynthesized and natural *B. napus* (3% and 4% of cells contained one univalent in resynthesized lines compared to 4% of cells in Stellar). These univalents were observed only among chromosomes A1, C1, A4, and A5. No A–C bivalent or univalent was detected in cultivar DH12075.

Nonhomologous 45S rDNA locus-containing chromosomes were associated in resynthesized *B. napus*, Stellar, and DH12075 (Figure 3 and Supplementary Figure S2). In many cases 45S rDNA-containing chromosomes formed a large conglomerate dominated by 45S ribosomal DNA signals (14% in S_1_ generation and 5.8% in S_11_ generation; Figure 3A) in resynthesized lines. Association among nonhomologous centromeres was also occasionally detected (Figure 3B). In Stellar, 45S rDNA locus-containing chromosomes did associate but never formed a single large conglomerate as in resynthesized lines (Figure 3C). A3 and C7 contain segmental homoeology according to genetic mapping (Parkin *et al.* 2005) and cytogenetic studies (Xiong *et al.* 2011). Associations between these two chromosomes were found in 5% of meiotic cells in Stellar among the 45S loci instead of along the chromosome arms. Compared the 45S rDNA locus associations at diakinesis, all the seven 45S rDNA locus-containing chromosomes showed significant higher frequencies of 45S rDNA associations in resynthesized line EL500 at S_11_ generation than those in Stellar (Supplementary Figure S2). Cultivar DH12075 had six pairs of 45S rDNA locus-containing chromosomes, which also showed significantly lower frequency of 45S rDNA associations than those of resynthesized line EL500 (Supplementary Figure S2). Association among nonhomologous 5S rDNA locus-containing chromosomes and nonhomologous centromeres was also occasionally observed (Figure 3, C and D).

These results indicate that the main meiotic irregularities observed during diakinesis in resynthesized allopolyploids were pairing among highly syntenic sets of homoeologous chromosomes and unresolved associations among 45S rDNA locus-containing chromosomes. Next, we examined how these irregularities impacted chromosome segregation at telophase I.

### Meiotic telophase I analyses in resynthesized and natural allopolyploid *B. napus*

Telophase I was analyzed in resynthesized line EL500 at both the S_1_ and S_11_ generation (Table 1) and in resynthesized lines EL1200 and EL5200 at the S_1_ generation (Supplementary Table S4), as well as among four *B. napus* cultivars (Table 1). All chromosomes could be unambiguously identified in the two daughter cells and were distributed in a mirror image relative to one another, allowing us to assess chromosome pairing and segregation patterns. Irregular chromosome segregation in telophase I led to the production of unbalanced daughter cells (nonadditive for parental haplotypes) (Table 1). In resynthesized lines, the average proportion of abnormal segregation events did not significantly decrease from the S_1_ generation (49%) to S_11_ generation (34%) (Table 2; two sample test of equal proportions, *P* = 0.187). Imbalanced telophase I daughter cells were also observed in natural cultivars at a rate of 8–12% (average 9%), and this was significantly lower than the rates in resynthesized *B. napus* (two sample tests of equal proportions, *P* < 0.05 with Bonferroni correction).

**Table 1:** Abnormal chromosome segregation detected in telophase I cells in *Brassica napus*

Chr^a^	EL500S1 Rep 1	EL500S1 Rep 2	EL500S11 Rep 1	EL500S11 Rep 2	Darmor	DH12075	Yudal	Stellar	Total from natural cultivars^b^
A1	5(5)	10(7)	6(5)	3(1)	2(1)	0	1(1)	0	3(2)
A2	3(2)	1(1)	1(1)	0	0	0	1(1)	1	2(1)
A3	4	3	0	2	0	0	0	0	0
A4	1	3	5	1	0	0	0	0	0
A5	1	4(1)	0	2	0	1	0	0	1
A6	1	0	1	0	0	0	1	0	1
A7	0	1(1)	0	0	0	0	0	0	0
A8	0	0	0	0	0	0	0	1	1
A9	3(3)	3(1)	2	6 (3)	1	1	0	0	2
A10	1	4(2)	2(1)	1	0	0	1	0	1
C1	6(5)	8(7)	5(5)	2(1)	1(1)	1	1(1)	0	3(2)
C2	2(2)	1(1)	1(1)	0	0	1	1(1)	0	1(1)
C3	1	0	0	1	0	0	0	0	0
C4	1	0	0	1	0	0	0	0	0
C5	3	2(1)	1	1	0	0	0	1	1
C6	1	3(1)	1	1	0	0	0	0	0
C7	1	1	0	0	0	0	0	1	1
C8	2(1)	2(1)	0	3	0	0	0	1	1
C9	5(2)	4(2)	2(1)	7(3)	1	0	0	1	1
Total	41(20)	50(26)	27(14)	31(8)	5(2)	4	6(4)	6	19(6)

Counts represent the number of telophase I cells (out of 50 pairs of cells analyzed in each plant) for which abnormal segregation was detected. The numbers inside parentheses represent the number of abnormal segregations that involved homoeologous substitution (see Xiong *et al.* 2011).

a Chromosome number.

b The total number of the abnormal chromosome segregations from four natural cultivars.

**Table 2:** Segregation of chromosomes in telophase I among resynthesized and natural allopolyploid *Brassica napus*

Behavior	EL500S1 Rep1	EL500S1 Rep2	EL500S11 Rep1	EL500S11 Rep2	Darmor	DH12075	Yudal	Stellar	Cultivar mean	*t*-Test^a^ (*P*-value)
Normal^b^	56	46	72	60	92	92	92	88	91	0.00832
Abnormal^c^	44	54	28	40	8	8	8	12	9	0.00832
19:19^d^	14	22	14	12	2	0	4	0	1.5	0.005108
18:20^e^	22	22	10	22	4	6	2	6	4.5	0.01407
Sister seg^f^	4	14	4	4	2	2	2	6	3	0.2598
A2/C2^g^ exchange	8	8	0	2	0	0	0	0	0	0.117
Fragment^h^	10	8	6	8	8	6	8	8	8	0.6806

**Figure 4:**
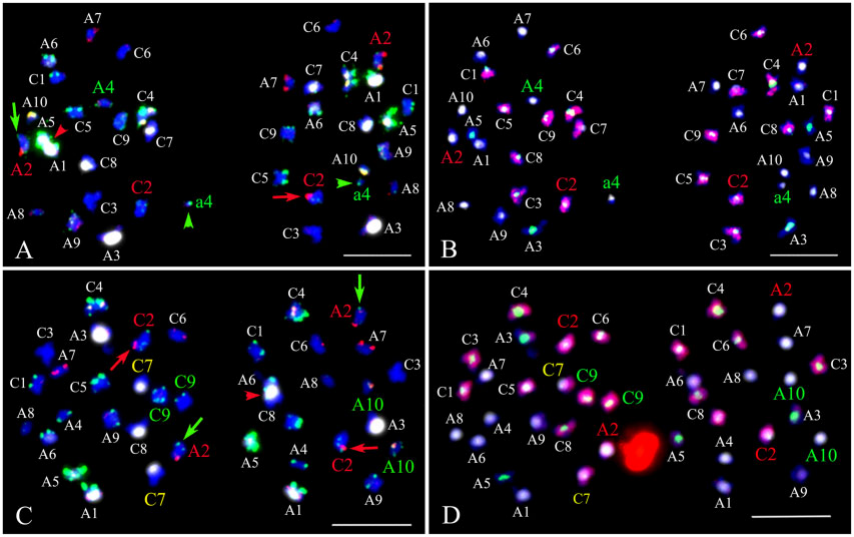
Homoeologous reciprocal exchanges, homoeologous chromosome replacement, early sister chromatid segregation, and 45S rDNA association detected at telophase I of resynthesized *Brassica napus*. Hybridizations were first performed using probe mixture 1 (panels A and C), and reprobed with mixture 2 (panels B and D) allowing unambiguous identification of all the parental chromosomes at telophase I. (A) In these telophase I cells, homoeologous reciprocal translocations between A2 and C2 were identified (green and red arrows). Chromosome A2 and C2 are homoeologous chromosomes. Normal A2 containing two sister chromatids had two red signals from BAC KBrH092N24 on long arms and weak green signal on short arm, and normal C2 had green signals on both arms. Note that one A2 (green arrow) lost one red signal on one chromatid, while one C2 (red arrow) gained one red signal on one chromatid. A4 homologs were unpaired (denoted with A4) and one homolog migrated to the leftmost pole and the other underwent early sister chromatid separation (denoted with green arrowheads and lower case a4). 45S rDNA association was detected between chromosomes A5 and A1 in the leftmost pole. (B) Same cells of A were re-hybridized using probes mixture 2, in which red signal from C-genome specific probe showed chromosomes from C-genome, and white and green signals from CentBr1 and CentBr2, respectively, showed the constitution of centromeres. (C) In this example, both sets of A2 and C2 homoeologs underwent reciprocal exchanges and migrated to opposite poles (green and red arrows). Again, note that the chromatids on these two sets of chromosomes are heteromorphic for red signals. Homoeologous chromosome replacement occurred between A10 and C9 (green text), and one C9 chromosomes lost green signal and one A10 gained green signal on long arm, indicating homoeologous pairing and exchange before telophase I. Homologous chromosome nondisjunction resulted in two C7 chromosomes migrating to one daughter cell (yellow text). 45S rDNA association was detected between chromosomes A6 and C8 in the rightmost pole. (D) Same cells of C were re-hybridized using probes mixture 2 to use for identification chromosomes. Scale bar = 10 μm.

Values are percentages out of 50 cells in each plant.

a Statistical analysis was performed between resynthesized lines and four natural allopolyploid *B. napus*.

b Normal segregation of parental chromosomes into daughter cells.

c % of daughter cells that were not additive for parental chromosomes (this includes abnormal segregation described footnotes d–f). Other abnormal, but complicated chromosome ratios are part of this value but are not summarized in this table.

d Nineteen chromosomes segregated to each daughter cell, but they were not additive for parental haplotypes.

e Unbalanced chromosome complement in daughter cells.

f Sister chromatids separated prematurely and segregated to daughter cells.

g A2–C2 homoeologous recombination detected (see Figure 4).

h Chromosome fragments detected.

In resynthesized *B. napus*, we observed homologous chromosome nondisjunction, homoeologous translocation, homoeolog replacement, early sister chromatid separation, and chromosome breakage (Figure 4 and Table 2). Chromosomes A1 and C1 demonstrated irregular chromosome segregation in an average of ∼14.5% and ∼8% of cells in the S_1_ and S_11_ generations, respectively (Table 1). Chromosome A8 displayed normal chromosome segregation in all cells of three resynthesized *B. napus* lines including EL500, EL1200, and EL5200 (Table 1 and Supplementary Table S4). Most chromosomes exhibited normal segregation among the four cultivars, but A1 and C1 demonstrated irregular segregation in an average of 1.5% of cells (Table 1). Most cases of irregular segregation involved nondisjunction, in which homologous chromosome pairs moved into the same daughter cell (Figure 4 and Supplementary Figure S3).

**Figure 5:**
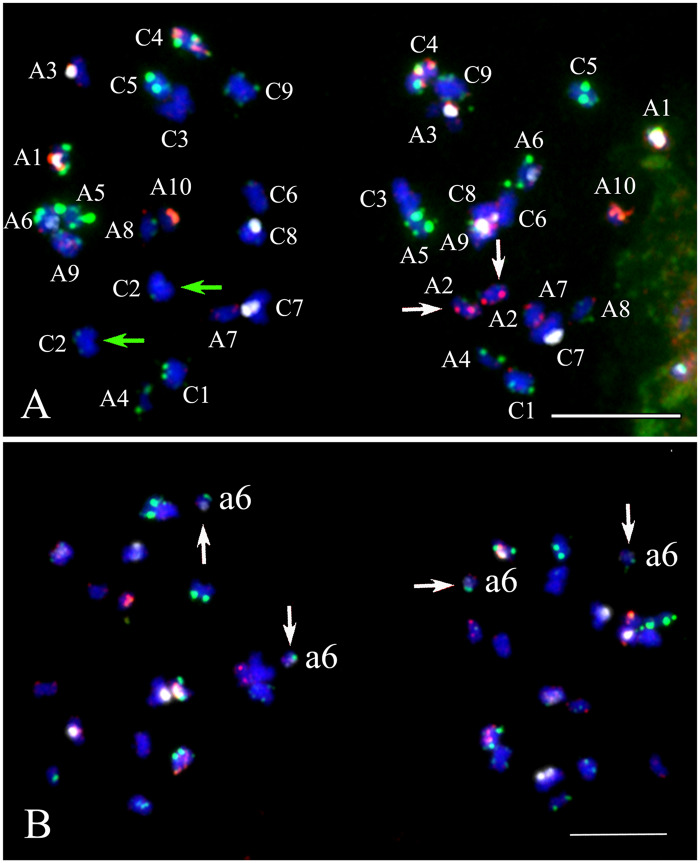
Homoeologous chromosome replacement and early sister chromatid separation *Brassica napus* cultivar Darmor. These hybridization results were obtained with probe mixture 1 (see *Materials and methods*). The same cells after hybridization with probe mixture 2 did not show here. (A) Homoeologous replacement among A2 and C2 homoeologs (white and green arrows, respectively). (B) Early sister chromatid separation of chromosome A6 (labeled with lowercase letter and white arrows). Scale bars = 10 μm.

Homoeologous chromosome replacement was common between chromosomes with high levels of conserved synteny. The highest frequency of homoeologous chromosome replacement was observed between chromosomes A1 and C1 (Table 1 and Supplementary Figure S3A). For A1, 80% (12 of 15 total cases for S_1_ replicates) and 67% (six of nine total cases for S_11_ replicates) of cases of irregular chromosome segregation involved homoeologous replacement with C1 (Table 1). For C1, 86% (12 of 14 total cases for S_1_ replicates; six of seven total cases for S_11_ replicates) of cases of irregular chromosome segregation involved homoeologous replacement with A1 (Table 1). A2 and C2 also demonstrated high rates of homoeologous chromosome replacement (Table 1). Chromosome replacement was also observed among homoeologous chromosomes retaining partial synteny, including: A4 and C4, A9 and C8, A9 and C9, and A10 and C9, but the frequencies were low in comparison (Table 1). Homoeologous replacement among A1, C1, A2, and C2 were seen infrequently in Damor and Yudal (Table 1 and Figure 5A).

Similar results were observed among the three resynthesized lines EL500, EL1200, and EL5200 (Supplementary Table S4), with homologous chromosome replacement being most common irregularity observed (Table 1 and Supplementary Table S4). The rates of imbalanced telophase I daughter cells in EL500, EL1200, and EL5200 were 49%, 56%, and 52%, respectively. In all three lines, chromosomes A1 and C1 demonstrated the highest rates of irregular segregation (Supplementary Table S4).

### Premature separation of sister chromatids during meiosis I

Early sister chromatid separation was observed at a low frequency in resynthesized *B. napus* among chromosomes A1, A3, A4, A5, A9, A10, C1, C6, and C9 (Figure 4A and Supplementary Table S5). Early sister chromatid separation occurred in one or both of the two homologs, and the two separated sister chromatids entered either the same or the different daughter cells. Among A-genome chromosomes, the average rate of early sister separation was 6% in the S_1_ generation and 4% in the S_11_ generation (Supplementary Table S5). Early sister separation in the C-genome was detected only in replicate 2 of the S_1_ generation (6% of telophase cells). Among the four *Brassica* cultivars surveyed, early sister chromatid separation was detected among chromosome A5, A6, A8, C7, C8, and C9 (see example in Figure 5B and Supplementary Table S3). It was detected only once (2% of cells) in Darmor, DH12075, and Yudal, and three events (6% of cells) were detected in Stellar (Table 2). Approximately 50% of the cases in which a chromosome underwent early sister separation the chromosome was unpaired with a homolog. In 17 out of 19 cases of early separation, the chromatids segregated to opposite poles. There was no obvious pattern of chromosomes showing early sister separation among resynthesized lines and natural cultivars. In addition, no significant difference in the frequency of early sister separation was found between resynthesized lines and natural cultivars (Table 2, *P*-value 0.259; *t*-test).

### Homoeologous reciprocal rearrangement and segregation in resynthesized *B. napus*

Homoeologous chromosomes A2 and C2 were the only homoeologous pairs in which rearrangements (*i.e.* homoeologous exchanges) could be detected utilizing our karyotype analysis approach. Signal heteromorphy of BAC clone KBrH092N24 on homoeologous chromosomes A2 and C2 permitted cytological verification of homoeologous chromosome exchanges at the positions between red signals KBrH092N24 and centromere (Figure 4 and Supplementary Figure S3). When recombination between homoeologous chromosomes occurred between A2 and C2, one sister chromatid on C2 acquired KBrH092N24 signals, and the reciprocal event was detectable as green signals from C2 on one sister chromatid of A2 (Figure 4 and Supplementary Figure S3). We carefully examined homoeologous exchanges in two resynthesized lines (EL500 and EL1200). In EL500, a total of 10 cases of homoeologous rearrangement between chromosomes A2 and C2 were observed in S_1_ and S_11_ generations. The average rate of homoeologous rearrangement between chromosomes A2 and C2 did not significantly decrease from the S_1_ generation to the S_11_ generation (Table 2; two sample test of equal proportions, *P* = 0.228). In the S_1_ generation, we found four cases of homologous rearrangement in each replicate (Table 2). In five cases, the recombinant A2 and C2 chromosomes clearly migrated into opposite daughter cells (Figure 4, A and B and Supplementary Figure S3, A and B), and in two cases they migrated to the same pole in telophase I (Supplementary Figure S3, C and D). In one case, homoeologous chromosome rearrangements occurred between both sets of A2 and C2 chromosomes (Figure 4, C and D). In the S_11_ generation, we found just two cases of homoeologous rearrangement between A2 and C2. Similarly in EL1200, five total cases of A2–C2 rearrangement were observed (Supplementary Figure S5). In two of these cases, recombinant A2 and C2 chromosomes migrated into opposite daughter cells (Supplementary Figure S5, A and E), in two cases they migrated to the same pole (Supplementary Figure S5, B and C), and in one case rearrangements occurred between both sets of A2 and C2 chromosomes (Supplementary Figure S5D). Thus, across the two resynthesized lines, rearrangement between the red signals of KBrH092N24 and the centromeres of A2 and C2 was observed in 5.7% (17 events in 15 cases out of 300 telephone I cells). In four natural *B. napus* cultivars, we did to detect homoeologous chromosome rearrangements in over 200 telophase I PMCs using these probes.

### Chromosome breakage and 45S rDNA loci translocation detected in telophase I

**Figure 6:**
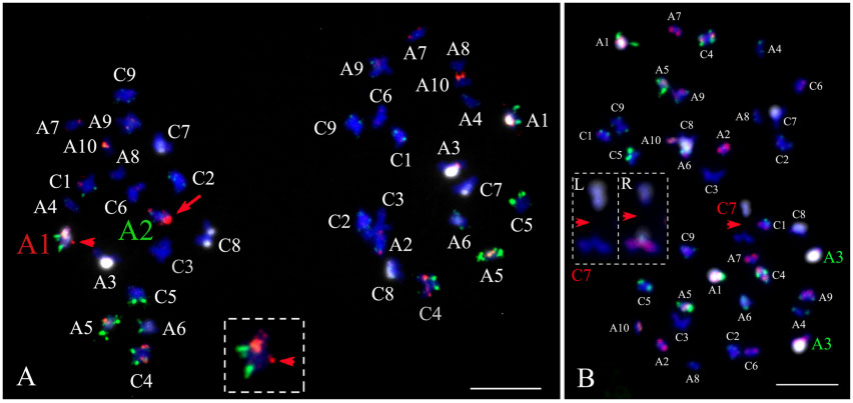
Chromosome breakage and 45S rDNA translocation in resynthesized *Brassica napus.* (A) 5S and partial 45S rDNA loss on one chromatid of A1 (red arrowhead) translocation to one chromatid of A2 (full size red arrow). Inset shows the A1 chromosome that experienced the loss with the Cy5 channel (white) removed. (B) Chromosome breakage at the centromere position of C7 (red arrowheads). Inset left (L) shows zoom in of C7 and inset right (R) shows the breakage at centromere position (white signal and red arrowhead) using probe mixture 2. Scale bars = 10 μm.

Chromosome fragments were observed in meiotic telophase I cells of resynthesized *B. napus* (Table 2, Figure 6, and Supplementary Figure S4A). Chromosome A1 contains 45S and 5S rDNA loci at interstitial positions on the long arm and two cases of chromosome breakage occurred at these loci, and in one case the 45S rDNA signals appeared to have translocated to A2 (Figure 6A). In one instance, breakage within a centromere was detected on chromosome C7 (Figure 6B). In one case, most of 5S, part of the 45S, and part of the centromere of one chromatid of A1 were lost (Supplementary Figure S4, A and B). One instance of chromosome breakage at a 5S locus was found on chromosome C4 (Supplementary Figure S4D). No rDNA breakages were detected among the natural cultivars; however, chromosome fragments were detected in telophase I daughter cells among all four *B. napus* cultivars and the average frequency was ∼8% (Table 2). This ratio was the same as that observed in resynthesized *B. napus*.

## Discussion

Meiosis in newly formed polyploids has been investigated by numerous scientists who have concluded that “meiotic irregularities” contribute to genome instability (Madlung *et al.* 2005; Lim *et al.* 2008; Szadkowski *et al.* 2010). In this study, we provide direct cytological evidence for homoeologous chromosomes pairing as tetravalents, exchanges, and segregation (including double reduction). We also observed unresolved pairing of 45S rDNA-containing chromosomes leading to chromosome breakage and non-homologous centromere associations. These direct cytological observations are very likely to be the cause of genetic changes and genome instability that we have previously reported on in our population of resynthesized *B. napus* (Gaeta *et al.* 2007; Xiong *et al.* 2011). These non-homologous associations correlated with the degree of known synteny between specific sets of homoeologs. Although observed, we did not find widespread A–C bivalents or premature sister chromatid separation as indicated in other reports. We also observed breakage at 45S rDNA loci, non-homologous centromere association, and early chromatid separation.

### Meiotic irregularities in resynthesized allopolyploid *B. napus*

Using our karyotyping method (Xiong and Pires 2011), we could unambiguously identify each of the 38 chromosomes of PMCs at diakinesis and telophase I in *B. napus* and were thus able to investigate the main “meiotic irregularities” in the newly formed polyploids. At diakinesis, the most common detected meiotic pairing irregularities were nonhomologous association which occurred among homoeologous chromosomes, 45S rDNA loci-containing chromosomes, and centromeres. Consistent with previous data, the occurrence of multivalents was an obvious meiotic deviation observed in our resynthesized polyploids (Lim *et al.* 2008; Chester *et al.* 2012). Nonhomologous associations were observed at high rates for both resynthesized and natural lines; however, counts across chromosomes were skewed in Stellar toward highly syntenic chromosomes (Figure 2A). This bias was much clearer when we separated homoeologous pairing from other observed associations (Figure 2B).

In resynthesized *B. napus*, high frequencies (about 15–37.5%) of cells with univalent A and C chromosomes were previously reported by Szadkowski *et al.* (2010) using BAC-FISH. In addition, A–C bivalents were observed in 30–47.5% of PMCs (Szadkowski *et al.* 2010, 2011). In contrast, in our analysis of resynthesized lines and the cultivar Stellar, we found univalents and A–C bivalents were much more rare (3–4%, and <4%, respectively). The difference between our observations and previous reports (Szadkowski *et al.* 2010, 2011) could be due to different synthetic *B. napus* lines studied and distinctly different stages of meiosis analyzed. Although diakinesis and metaphase I of meiosis were the common stage for counting the frequency of bivalents and multivalent (Grandont *et al.* 2014), at the stage of diakinesis in which we studied meiosis, some of these types of pairing anomalies would have been resolved by metaphase I. Additionally, we utilized an improved karyotyping method (Xiong and Pires 2011) and the materials selected for meiotic analysis were “intact” euploids that were additive for all chromosomes from parental subgenomes. Furthermore, we provide evidence for abnormal chromosome segregation during telophase I in resynthesized *B. napus*. High frequencies (approximately 28–54%) of daughter cells in telophase I of S_1_ and S_11_ generations were non additive for parental chromosomes, which was significantly higher than observations in cultivated *B. napus* (Table 2). Our cytogenetic data demonstrates for the first time that the most common abnormal chromosome segregation pattern in telophase I was homoeolog replacement (Table 1), which likely resulted from multivalent associations and random segregation of homoeologs. Such unbalanced products following telophase are not necessarily expected to result in viable gametes and progeny; however, their occurrence in PMCs may explain our previous observations of homoeologous chromosome replacement and compensation (Xiong *et al.* 2011).

### Disomic, polysomic, and intermediate inheritances depend on the degree of homoeologous synteny in resynthesized *B. napus*

In this study, cytogenetic analysis of meiosis in the resynthesized *B. napus* revealed that homoeologous pairing, segregation, and inheritance varied among different chromosomes. Chromosomal inheritance patterns were highly related to the degree of the synteny between homoeologous chromosome pairs. Homoeologous chromosomes (like A1/C1 and A2/C2) retaining entire length synteny (Parkin *et al.* 2005; Chalhoub *et al.* 2014) showed polysomic inheritance suggested by our observation of frequent tetravalent formation at diakinesis (Figure 2), resulting in random homoeologous chromosome segregations in telophase I (Supplementary Figure S3A). This result was consistent with the observation of tetrasomic inheritance of A2/C2 in *B. napus* using RFLPs in the mid-nineties (Parkin *et al.* 1995). In contrast, chromosome A8, which contains only small regions of collinearity with C3 or C8 (Chalhoub et al. 2014), paired exclusively with its homolog (Figure 2) and segregated normally (Table 1 and Supplementary Table S4). Intermediate inheritance, referring to inheritance patterns intermediate between disomic and polysomic, was also observed among chromosomes sharing partial collinearity between homoeologs. These results suggest that pre-existing chromosomal restructuring between parental genomes had a great impact on the inheritance and chromosome stability in resynthesized *B. napus*.

### Reciprocal homoeologous chromosome replacements, exchanges, and double reductions in resynthesized *B. napus*

Different models of homoeologous recombination and segregation in resynthesized allotetraploids and autotetraploid have been proposed in previous molecular marker and cytogenetic studies (Hufton and Panopoulou 2009; Gaeta and Pires 2010; Parisod *et al.* 2010; Szadkowski *et al.* 2010). Gaeta and Pires (2010) presented a model in which the homoeologous chromosomes that recombine segregate to opposite poles. This assumption was based on the rate at which reciprocal forms of translocations were detected in the homozygous form in progeny of self-pollination. The observation of frequent A–C bivalents in metaphase I led Szadkowski *et al.* (2010) to suggest gametes with a normal chromosome numbers could carry two copies of A1(with no C1) or two copies of C1 (with no A1), suggesting a model whereby homoeologous bivalents lead to nullisomic/disomic gametes.

In our study, we found that homologous chromosomes generally paired as bivalents or mispaired with homoeologous chromosomes to form tetravalents of A1–C1 or A2–C2, rather than homoeologous bivalents (Figure 2B). A very low frequency (less than 2% for each) of univalent or A–C bivalent was detected for these four chromosomes in PMC. At telophase I, homoeolog replacements between A1 and C1, as well as between A2 and C2 were the main chromosomal segregation irregularity, indicating that homoeologs replacement might be the main pathway to induce nullisomic/disomic gametes in resynthesized *B. napus*.

Homoeologous cross-overs between the red signal of KBrH092N24 and the centromere at the long arms of A2 and C2 could be detected utilizing our karyotype analyses. In telophase I of resynthesized *B. napus*, we observed 17 events of segregating homoeologous translocations among 300 PMCs resulting from the recombinations between A2 and C2 (Figure 4 and Supplementary Figures S3 and S5). These data provided a visual verification for hypotheses that had been previously formulated based on the loss and duplication of molecular markers in resynthesized *B. napus* (Pires *et al.* 2004; Udall *et al.* 2005; Lukens *et al.* 2006; Gaeta *et al.* 2007; Gaeta and Pires 2010). We found that the recombined homoeologous fragments could segregate randomly either to the same or to different daughter cells. We also provided direct evidence of whole homoeologous chromosome replacement that could make the analysis of homoeologous chromosome recombination more complicated. In addition, two cases of homoeologous chromosome rearrangement occurred between both sets of A2 and C2 chromosomes making it difficult to determine the segregation pattern of the recombined homoeologs (Figure 4C and Supplementary Figure S5C).

Double reduction is a peculiarity of polysomic inheritance in which sister chromatid fragments segregate into the same gamete during meiosis (Butruille and Boiteux 2000; Huang *et al.* 2019). In S_1_ resynthesized *B. napus*, four cases the recombinant sister chromatid fragments (red signals) between homeologs A2 and C2 migrated to the same pole in telophase I (Supplementary Figure S3C) were observed in EL500 and EL1200. Therefore, we provide direct evidence for double reduction induced by crossing-over between non-sister chromatids between homoeologs.

### Nonhomologous association among centromeres and 45S rDNA locus-containing chromosomes

In pachytene, nonhomologous centromere associations occurred and only 5–6 centromere foci were visible in both natural and resynthesized plants. As meiosis progressed, these nonhomologous centromere associations largely disappeared in natural lines. This is consistent with observations in monocots and mammals that suggest early meiosis involves nonhomologous centromere associations that are sorted out during the progression of meiosis (Church and Moens 1976; Stewart and Dawson 2008). Compared to those in cultivated *B. napus*, the residual nonhomologous centromere association was more visible in resynthesized lines at diakinesis. At telophase I, one case centromere breakage was observed in resynthesized *B. napus*. In previous experiments 7.9% of 38 polyploids lines contained somatic karyotype changes that involved rearrangements and chromosome breakage within centromeres (*i.e.* allo- and auto-syndetic chromosome rearrangements; Xiong *et al.* 2011). Polyploidy induced centromere association has been reported in within the *Triticeae* (Martinez-Perez *et al.* 2000).

45S rDNA locus-containing chromosomes were frequently associated at diakinesis in both resynthesized and cultivated *B. napus*; however, significantly higher frequencies of 45S rDNA associations among the seven 45S rDNA locus-containing chromosomes were observed in resynthesized lines (Supplementary Figure S2). Association of 45S rDNA loci was expected because of the role of these chromosomes during the formation of the nucleolus (McClintock 1934; Pecinka *et al.* 2004). Xiong *et al.* (2011) analyzed a population of 38 replicate *B. napus* allopolyploids and found that 21% of the lines contained karyotype changes directly due to 45S rDNA rearrangements and deletions. Certainly, rDNA changes are a common occurrence in other allopolyploids, including *Arabidopsis* (Pontes *et al.* 2004), *Nicotiana* (Kovarik *et al.* 2004), *Hepatica* (Weiss-Schneeweiss et al. 2007), and *Tragopogon* (Lim *et al.* 2008; Malinska *et al.* 2010).

Taken together, both nonhomologous centromere and 45S rDNA associations at early meiosis are normal phenomenon in plants (Martinez-Perez *et al.* 2000; Pecinka *et al.* 2004). Nonhomologous centromere and 45S rDNA associations were resolved mostly at diakinesis in natural *B. napus*, while those in resynthesized *B. napus* were entangled together. Therefore, here, we considered the phenomena of high ratio of nonhomologous centromere and 45S rDNA associations at diakinesis as a kind of “meiotic irregularity” in resynthesized allopolyploid. Our results suggested that the unsolved 45S rDNA association and nonhomologous centromere at diakinesis may induce the breakage or rearrangements at 45S rDNA loci and centromere positions in resynthesized *B. napus*.

### Comparisons of the chromosome behaviors during meiosis I between resynthesized and natural *B. napus* and inferring the genome stabilization of natural *B. napus*

In most established polyploids, including *B. napus*, meiotic instability is rare or absent, which shows a predominantly diploid-like meiosis with bivalents forming at metaphase I and disomic inheritance (Udall *et al.* 2005; Grandont *et al.* 2014). Our cytological observations showed that early during pachytene, no clear tetravalents were observed in natural *B. napus*, while synaptic tetravalents showing pairing partner switches between homoeologous chromosomes were frequently observed in resynthesized *B. napus*. In resynthesized *B. napus*, tetravalents between homoeologous chromosomes persisted to diakinesis, which were linked to incorrect segregations. These results demonstrated that early in prophase I, homoeologous chromosome pairing was largely suppressed in natural *B. napus* compared with those in resynthesized *B. napus* (Figure 2B).

In addition, we speculate that the successful resolution of non-homologous centromeres and 45S rDNA associations must be essential to the process of meiotic stabilization in natural *B. napus*. By contract, our resynthesized lines lacked the ability to successfully resolve these mispairings, leading to chromosome breakage and rearrangement at centromeres and rDNA positions (Supplementary Figure S4). Consistent with previous reports (Osborn *et al.* 2003b; Parkin *et al.* 2005; Piquemal *et al.* 2005; Udall *et al.* 2005; Howell *et al.* 2008), our results supported that cross-overs among homoeologous chromosome pairs were largely suppressed in Stellar. We found 44.5% of homoeologous pairing among A1 and C1 at diakinesis in Stellar. In Stellar we did observe that 44.5% of cells showed A1–C1 homoeologous pairing in diakinesis, but we did not detect abnormal segregation in telophase I (Table 1). Similarly we observed homoeologous pairing between A2 and C2 in diakineses that did not result in rearrangement. Therefore, genomic stabilization in natural *B. napus* probably involves reduction in homoeolog pairing and cross-over and successful resolution of centromeres and 45S rDNAs.

### Meiotic genome restructuring over 11 generations in resynthesized *B. napus*

Recombination among homoeologs is detectable in the S_0:1_ generation and perpetuates for at least a dozen generations following resynthesis (Parkin *et al.* 1995; Lukens *et al.* 2006; Gaeta *et al.* 2007; Gaeta and Pires 2010). Szadkowski *et al.* (2010) described the first round of meiosis of resynthesized *B. napus* as a “genome blender,” a phenomenon that Gaeta and Pires (2010) argued can lead to a “polyploid ratchet.” Unlike *Brassica* allotetraploids, wheat allotetraploids display more genomic changes immediately after hybrid formation when compared to later generations selfing progeny (Feldman *et al.* 1997; Shaked *et al.* 2001).

The average proportion of abnormal daughter cells decreased from 49% in the S_1_ generation to 34% in S_11_ generation. The average rate of homoeologous rearrangements between chromosomes A2 and C2 decreased from 8% in the S_1_ generation to 1% in the S_11_ generation. These data may suggest a pattern of genome stabilization over time, but the differences were not statistically significant, possibly due to the number of replicates analyzed. Ha *et al.* (2009) suggested that genome stability increased over successive generations in resynthesized *Arabidopsis* allopolyploids through the establishment of stable siRNA expression patterns.

In summary, cytogenetic analyses to differentiate all chromosomes at meiotic diakinesis and telophase I were performed in resynthesized and natural allopolyploids *B. napus* using FISH. In resynthesized *B. napus*, the main meiotic errors inducing the meiotic instability were homoeologous association, unresolved 45S rDNA association, and nonhomologous centromere association at diakinesis. Abnormal chromosome segregation included homoeologous chromosome replacement, homoeologous translocation, and 45S rDNA breakage. Different chromosomal inheritances including strict disomic, intermediate, and polysomic inheritance were discovered in resynthesized *B. napus* and the inheritance patterns were related to the collinearity between homoeologous chromosomes.
